# Assessing the adoption and application of the Namibian biosafety labelling regulations and determining their impact on Namibian food and feed importers

**DOI:** 10.3389/fbioe.2023.1224992

**Published:** 2023-07-06

**Authors:** Paulus Mungeyi, Percy Maruwa Chimwamurombe, Grace Nandesora Kangueehi

**Affiliations:** ^1^ Department of Biology, Chemistry and Physics, Namibia University of Science and Technology, Windhoek, Namibia; ^2^ Department of Agricultural Sciences and Agribusiness, Namibia University of Science and Technology, Windhoek, Namibia

**Keywords:** biosafety, GMOs, GM products, importers, labelling

## Abstract

The study was carried out to investigate the implications of the Namibian biosafety regulations on Namibian food and feed importers. After the Biosafety Act, 2006 (Act No. 7 of 2006), the biosafety regulation was gazetted in 2016, which saw the implementation of the national framework, the impact of food and feed importers was not known. The objective of the study was to assess the adoption and application of the national biosafety labelling regulations by food and feed importers. In addition, the impacts of these regulations on Namibian food and feed importers were assessed. The study used a structured online and hard copy survey questionnaire based on responses from 340 Namibian importers of food and feed products from eight identified Namibian regions: Khomas, Erongo, Kavango West, Kavango East, Omusati, Oshana, Ohangwena, Oshikoto, and Zambezi who have the knowledge required for the adoption and application of the Namibian biosafety labelling regulations. Using the Mann-Whitney test, the study confirmed that individuals who are aware of the biosafety Act, 2006 (Act No. 7 of 2006) are less likely to agree with statements such as experiencing problems in fulfilling requirements under the biosafety regulations. It was further concluded that there is a need to reduce the current administrative burdens for handling applications and improve dialogue between regulators and the food and feed importing industry while increasing the competence of regulators and creating more labelling regulation awareness for food and feed importers. The study further suggests that public awareness is required beyond food and feed importers.

## 1 Introduction

Food and feed consisting of, containing, or derived from GMOs have been circulating the globe since the beginning of the 21st century (Aguilera et al., 2013).

Many countries that are using GMOs and derived products have put in place biosafety regulations to ensure the safe handling, transport, and use of GMOs. During the last 15 years, more than 40 countries have approved labelling laws. However, the level of implementation of these labelling laws differs greatly from one country to another based on their characteristics, (Phillips and McNeill, 2000; Carter and Gruère, 2003; Haigh, 2004). Twardowski and Małyska (2015) stated that in countries that have biosafety regulatory frameworks in place, such as in the European Union (EU), the regulations are rather complicated and that leads to the slow approval process of genetically modified crops and products. In many African countries, these regulations are still lacking and making it impossible to approve the use of GMOs (Adenle et al., 2017). Kaur et al. (2018), have indicated that, out of 54 countries, the use of GMOs has only been approved in a few African countries like South Africa, Eswatini, Malawi, Kenya, Ethiopia, Sudan, Nigeria, and Burkina Faso. One of the requirements for regulation is the labeling of GM food products and processed products with the aim to give consumers choices (Twardowski and Małyska, 2015). General rules on food labelling can be divided into two categories, namely, rules on nutritional information and rules on labelling. The rules on labelling are associated with obligatory information for all food stuffs (Borges et al., 2018). Aarts et al. (2002) have further highlighted that GM products should be labelled to ensure traceability of these products at all stages of the food supply chain. The primary goals of these standards are to safeguard consumers’ health and safety, as well as to guarantee that food traders implement fair international and regional trading procedures. (Borges et al., 2018). One such standard is guided by Codex General Standard for The Labelling of Prepackaged Foods (Codex) which recommends applying a fair labelling rule including GM products considering risk assessment of such products (Borges et al., 2018). Over time, extensive literature has been developed on the importance of labelling food products. One emphasis was put on consumer health on which Twardowski and Małyska (2015) argued that labelling help customers in understanding the ingredients in terms of specific doses and/or spotting ingredients that may cause allergies. Kedisso et al. (2022) argue that labelling is important since it is a tool that regulators employ to guarantee that traders disclose information to consumers in hopes of decreasing scientific uncertainty and consumer arguments over the safety of GMOs.

As a result of the high degree of competition in the global food market, customer choice can have a considerable influence on the sort of product selected. Hence, consumer choice also involves distinguishing between GMO and non-GM food products (Albert, 2010). Hence, this is an indication that the buying behaviours of consumers’ are driven by health concerns associated with processing aids used in the production and manufacturing of the food products. Hu et al. (2021) further argued that consumers will choose GM, but only when it is significantly less expensive than non-GM. These findings are supported by Azila-Gbettor et al. (2013) who added that consumer choice becomes difficult to achieve in some regions of the world due to the diversity of products in the market and the ability of consumers to read a particular language due to low literacy levels. Therefore, when it comes to labelling of food products, Choi (2010) stressed that labelling based on the percentage threshold level of GM content may also be a barrier to trade. Thus, there is a lot of uncertainty in the implications of mandatory labelling regulations in terms of what requirements must be satisfied for food to be considered a GM product (MacFadden, 2017). Asioli et al. (2017) contend that, unlike in poor nations, consumers in advanced nations are more interested in knowledge about food production methods than in the ingredients of the food products they eat. Other barrier to trade in terms of consumer choice is associated by pricing. Thus, if looking at the angle of the cost implication of labelling, some proponents of mandatory labelling are of the opinion that food companies change labelling to reduce the cost associated with this effect McFadden (2017). Thus, researcher have therefore argued that when it comes to the labelling of food products, consideration should not only be based on national laws such as the biosafety laws, but great consideration should be put on the interpretation and align laws in line with the Word Trade Orgaisation agreements (Borges et al. (2018; Komen, 2012; Van der Walt, 2001).

Namibia now depends on imports to fulfill domestic demand since it cannot produce enough maize as most (over 70%) of the white maize grain imported now comes from South Africa (NAB, 2021). As outlined in the Biosafety Act of 2006, Namibia, like many other nations, has implemented legislation requiring the labelling of genetically modified food and feed items (Biosafety Act No. 7, 2006). All registered Namibian firms dealing with genetically modified foods or feed must mark them in accordance with the Act’s three 3) categories (separately or individually packaged raw agricultural commodity, Raw agricultural commodity, which is not separately or individually packaged and processed genetically modified food or feed). The regulation that allows for the labelling of GMOs and products under the Biosafety Act was gazetted on 1 November 2016. The labelling criteria for GM food and feed in Namibia is 0.9 percent, while in South Africa is 5%. According to Jacobs (2018), the Namibian labelling Biosafety regulations have made it more stringent and costly for South African products exporters to modified and label their packaging of products in line with Namibia’s, labelling regulation requirements. Other criticisms included the fact that Namibia’s GMO labelling threshold was impracticable and had a major negative impact on South African local grain exporters. This is an indication of regional trade barriers between Namibia and South Africa caused by the Biosafety Labelling Regulations. Trade-related regulations in developing countries in terms of the trend toward harmonizing regulations with regards to trade-related regulations of GM food have been advocated by Gruère (2006). Although the GM labelling requirements exist in some countries, they are often challenging to implement in others due to a variety of circumstances, including a lack of understanding among consumers and those who should follow the regulations, such as companies who import or export GMO-related products (Bain and Dandachi, 2014; Adalja et al., 2022). Several empirical studies have focused on investigating consumer perceptions of food security, genetically modified crops, and food safety (Albert, 2010; Aerni et al., 2011; Valente and Chaves, 2018). Unfortunately, the existing research has been limited in assessing the overall implications of GMO labelling on producers, processors, and importers. No study was conducted in Namibia to assess the impact of the national biosafety labelling regulation on producers, processors, and importers after enactment. Hence the necessity to critically investigate the implications of the Biosafety Act, 2006, considering the trading partners.

## 2 Materials and methods

Assessing the adoption and application of the Namibian Biosafety labelling regulations and determining their impact on Namibian food and feed importers was undertaken using a quantitative research approach. The response to the assessment were based on data collected through a structured questionnaire survey. The questionnaire survey was sent to 180 Namibian importers (importing food and feed products) based in most regions identified, namely Khomas, Erongo, Kavango West, Kavango East, Omusati, Oshana, Ohangwena, Oshikoto, and Zambezi who answered the survey from 28 April 2020 to 12 January 2021. A purposive sampling method was used to identify the respondents who were required to have basic knowledge on Namibian Biosafety labelling regulations adoption and application. A sample of experts comprised of the following groups: Business Owner, Executive Management, Middle Management, Junior Management and Junior Staff were surveyed. Contextual information regarding the background of the respondents is shown in table 1.

**TABLE 1:** Information on the respondents’ context.

Sample	N	%
Gender		
Female	90	66.7
Male	45	33.3
No of years in the food and feed industry		
0–5 Years	30	22.2
6–10 Years	29	21.5
>10 Years	76	56.3
Position of the respondent		
Business Owner	43	31.9
Executive Management	20	14.8
Middle Management	59	43.7
Junior Management	8	5.9
Junior Staff	5	3.7
Full-time employees in the company		
0–20	49	36.3
21–40	24	17.8
41–60	22	16.3
61–80	9	6.7
81–100	5	3.7
>100	26	19.3

Questions in the survey were technical and specific to the Namibian biosafety labelling regulations, therefore only those who have knowledge or have been involved in the adoption and application of the Namibian biosafety labelling regulations and their impact on the food and feed importers in Namibia were included, hence the public was excluded. Survey questions were answered based on a five-point Likert scale. For instance, Robayo-Avendaño et al. (2018) used a 3-point scale, Coşkun and Olhan, (2022) as well as Nowamukama (2022) used a 5-point scale while Shooshtari et al. (2022) used a 6-point scale to assess the awareness of GMOs in terms of the Iran Biosafety Act: case study of Tehran city. The five-point scale used in this study provided the required detail for the evaluation while reducing potential over-complication caused by a higher number of alternatives (e.g., a 6-point scale (Leung, 2011). The survey responses in this study were rated as follows: 1—Strongly Disagree (SD); 2—Disagree(D); 3—Neither Agree nor Disagree (NAD); 4—Agree A); 5—Strongly Agree (SA).

A total of 137 were responded, making it 76.1% of the response rate and only 135 were responded correctly making up 98.5% usefulness in the analysis. Thirteen (13) of the non-response were no more in business and 30 were either not interested or non-responding at all. Survey results were analysed using the Statistical Package for the Social Sciences (SPSS 26) and Microsoft Excel (2020). Descriptive statistic statistics were employed to tabulate data, cross tabulations, and Chi Square. According to Pimentel (2010), the five-point Likert scale is an interval scale with a highly significant mean of 1–1.8, which means strongly disagree, 1.81 to 2.60, disagree, 2.61 to 3.40, neutral, 3.41 to 4.20, agree, and 4.21 to 5, which means strongly agree. When Likert scale, items are considered to have an interval measurement, and the information for all respondents is frequently summarized in the form of a weighted mean. Following this technique, the resultant weighted mean was interpreted using an interval with a matching verbal explanation.

## 3 Results

This section explores the perceptions of various actors on substantive effectiveness of the Namibia Biosafety framework as presented under three elements: a) Biosafety Act awareness, b) The BSL adoption and application, c) Impact of labelling regulations and d) Eliminating trade barriers posed by the Biosafety labelling regulations.

### 3.1 Biosafety Act awareness

#### 3.1.1 Awareness of the existence of the Biosafety Act, 2006

Table 2 shows the perceptions of the survey respondents on the awareness of the existence of the Biosafety Act, 2006. The *z* value that was based on the Mann-Whitney Test ranks on the awareness of the existence of the Biosafety Act, 2006 is -2.328, with a significance level of *p* = 0.020. This result shows that since the *p*-value calculated is significant at 0.02, individuals who are aware of the existence of the Biosafety Act, 2006 are less likely to agree with the labeling of Genetically Modified Food and Feed (processed) as required by the Biosafety labelling regulations as compared to individuals who are not aware of the existence of the Biosafety Act, 2006.

**TABLE 2:** Mann-Whitney Test ranks on the awareness of the existence of the Biosafety Act, 2006.

Are you aware of the existence of the biosafety Act, 2006?		n	Mean rank	Sum of ranks
Yes		125	65.80	8,225.00
No		10	95.50	955.00
Total		135		
Test Statistics				
Mann-Whitney U	350.000			
Wilcoxon W	8.225E3			
Z	−2.328			
Asymp. Sig. (2-tailed)	0.020			

#### 3.1.2 Awareness of the labelling requirement under the biosafety Act, 2006

The Mann-Whitney Test was further run to establish whether individuals who are aware of the labelling requirements under the Biosafety Act, 2006 agree with the labeling of Genetically Modified Food and Feed (processed) as required by the Biosafety labelling regulations as compared to those whose companies are unaware. The *z* value is -1.101 with a significance level of *p* = 0. 271. The results shows that there is sufficient evidence to conclude that there is no significant difference in the agreeance of individuals who are aware of the labelling requirement under the Biosafety Act, 2006 with the labeling of Genetically Modified Food and Feed as required by the Biosafety labelling regulations in Table3.

**TABLE 3:** Mann-Whitney Test ranks on the awareness of the labelling requirement under the Biosafety Act, 2006.

Are you aware of the labelling requirement under the biosafety Act, 2006		n	Mean rank	Sum of ranks
Yes		98	65.74	6,442.50
No		37	73.99	2,737.50
Total		135		
Test Statistics				
Mann-Whitney U	1.592E3			
Wilcoxon W	6.442E3			
Z	-1.101			
Asymp. Sig. (2-tailed)	0.271			

### 3.2 The BSL adoption and application

The study sought to determine how biosafety labelling regulations are adopted and applied in Namibia. To achieve this objective, the study looked at general Biosafety Act, 2006 awareness and how it related to adoption before assessing the acceptance of biosafety labelling regulations. Twardowski and Małyska (2015) have indicated that stagnation in the implementation of biosafety frameworks is due to a lack of awareness.

By running the Mann-Whitney Test, the study confirmed the findings that individuals who are aware of the existence of the Biosafety Act, 2006, including those who are experiencing problems in fulfilling the requirements of the Biosafety Act, 2006, are less likely to agree with the labeling of Genetically Modified Food and Feed (processed) as required by the Biosafety labelling regulations. Responses of individuals from companies that have applied to fulfil the requirements of the Biosafety (Secretariat of the Convention on Biological Biodiversity, 2000) indicated that they are more likely to agree with the labeling of Genetically Modified Food and Feed (processed) as required by the Biosafety labelling regulationsin Table4.

**TABLE 4:** Mann-Whitney Test ranks companies applying to fulfill the requirements of the Biosafety Act, 2006.

Has your company applied to fulfil the requirements of the biosafety Act, 2006		N	Mean rank	Sum of ranks
Yes		73	75.71	5,526.50
No		62	58.93	3,653.50
Total		135		
Test Statistics				
Mann-Whitney U	1.700E3			
Wilcoxon W	3.654E3			
Z	-2.502			
Asymp. Sig. (2-tailed)	0.012			

#### 3.2.1 Companies applying to fulfil the requirements of the Biosafety Act, 2006

A Mann-Whitney test was run to assess whether individuals whose companies applied to fulfil the requirements of the Biosafety Act, 2006 agree with the labeling of Genetically Modified Food and Feed (processed) as required by the Biosafety labelling regulations compared to those whose companies are unaware. The *z* value is -2.502 with a significance level of *p* = 0.012. Based on the mean rank values observed in the ranks table, it can be concluded that individuals whose companies have applied to fulfil the requirements of the Biosafety Act, 2006 are more likely to agree with the labeling of Genetically Modified Food and Feed as required by the Biosafety labelling regulations as compared to individuals whose companies have not applied to fulfill the requirements of the Biosafety Act, 2006.

#### 3.2.2 Companies experiencing problems in fulfilling requirements of the Biosafety Act, 2006

Individuals whose companies are experiencing problems in fulfilling requirements of the Biosafety Act, 2006 are less likely to agree with labeling of Genetically Modified Food and Feed as required by the Biosafety labelling regulations in comparison to individuals whose companies are not experiencing problems in fulfilling requirements of the Biosafety Act, 2006 Table5.

**TABLE 5:** Mann-Whitney Test ranks companies experiencing problems in fulfilling requirements of the Biosafety Act, 2006.

Is your company experiencing problems in fulfilling the requirements of the biosafety Act, 2006?		N	Mean rank	Sum of ranks
Yes		54	85.28	4,605.00
No		81	56.48	4,575.00
Total		135		
Test Statistics				
Mann-Whitney U	1.254E3			
Wilcoxon W	4.575E3			
Z	-4.222			
Asymp. Sig. (2-tailed)	0.000			

### 3.3 Impact of labelling regulations

#### 3.3.1 Threats posed by the biosafety labelling regulations

##### 3.3.1.1 The most significant threats posed by labelling regulations to a company

As seen from the Likert scale mean in Table 6, the response for all the questions regarding the biggest threats posed on companies surveyed regarding labelling regulations falls in the range of (2.01–3.00). Thus, it can be concluded based on the range used that the average response recorded for all the questions were neutral. The responses were slightly leaning towards monopoly in the supply chain.

**TABLE 6:** The most significant threats posed by labelling regulations to a company.

Statements concerning the biggest threats posed to your company regarding labelling regulations		N	Minimum		Maximum	Mean	Std. Deviation
Increases Business costs		135	1		5	2.50	1.021
Increases foreign competition		135	1		5	2.70	1.093
Lengthy administration process		135	1		5	2.40	1.001
Increase in taxes and permit fees		135	1		5	2.61	1.100
Monopoly in the supply chain		135	1		5	2.79	1.153
Too many import laws		135	1		5	2.42	1.143
Lack of technical capacity of regulators		135	1		5	2.50	1.085
Limited understanding of the law by importers		135	1		5	2.50	1.139
Lack of uniformity in the Namibia biosafety regulations		135	1		5	2.63	1.028
Inadequate capacity to export products to markets		135	1		5	2.63	1.028
*c Note. 5 strongly agree (4.01–5.00), 4 agree (3.01–4.00), 3 neutral (2.01–3.00), 2 disagree (1.01–2.00), 1 strongly disagree (0.01–1.00)*							

Reliability Coefficient	Cronbach’s Alpha			N of Items			
0.912			10				

##### 3.3.1.2 Long-term consequences for businesses as a result of labelling regulations

Results show that out of the six long-term effects for businesses caused by labelling regulations, the threats posed by the Biosafety labelling regulations causes long term consequences such as reduction in production, revenues, increase in production costs, loss of markets, retrenchment of employees and closing of the company. As seen from the Likert scale mean in Table 7, the response for all the questions regarding the long-term consequences faced by companies if the biggest threats posed to their companies regarding labelling regulations are not resolved falls in the range of (2.01–3.00). Thus, it can be concluded based on the range used that the average response recorded for all the questions were neutral with responses leaning toward ‘Lead to loss of markets’.

**TABLE 7:** Long-term consequences for businesses as a result of labelling regulations.

Long-term consequences faced by companies if the biggest threats posed to your company regarding labelling regulations are not resolved		N	Minimum	Maximum		Mean	Std. Deviation
Reduction in production		135	1	5		2.47	0.853
Reduction in revenues		135	1	5		2.53	1.028
Increase in production costs		135	1	5		2.38	0.969
Lead to loss of markets		135	1	5		2.59	1.095
Lead to retrenchment of employees		135	1	5		2.46	1.091
Lead to closing of the company		135	1	5		2.41	0.988
*c Note. 5 strongly agree (4.01–5.00), 4 agree (3.01–4.00), 3 neutral (2.01–3.00), 2 disagree (1.01–2.00), 1 strongly disagree (0.01–1.00)*							

Reliability Coefficient	Cronbach’s Alpha				N of Items		
0.882				6			

##### 3.3.1.3 Policy recommendations for eliminating trade threats imposed by labelling regulations

The policy recommendations towards the elimination of trade threats imposed by labelling regulations looked at weather the policy should reduce regulatory administrative burden, increase the competence of regulators, improve regulatory monitoring processes, improve dialogue between regulators and industry or create more awareness of the regulation. As seen from the Likert scale mean in Table 8, the response for all the questions regarding what policymakers should do to remove the threats in question caused by labelling regulations to allow importing companies to remove trade barriers falls in the range of (2.01–3.00). Thus, it can be concluded based on the range used that the average response recorded for all the questions were neutral, however, respondents were more on ‘improve regulatory monitoring processes’.

**TABLE 8:** Policy recommendations for eliminating trade threats imposed by labelling regulations.

What policymakers should do to remove the threats in question caused by labelling regulations to allow importing companies to remove trade barriers		N	Minimum		Maximum	Mean	Std. Deviation
Reduce regulatory administrative burden		135	1		5	2.52	1.028
Increase the competence of regulators		135	1		5	2.25	1.020
Improve regulatory monitoring processes		135	1		5	2.46	1.131
Improve dialogue between regulators and industry		135	1		5	2.16	1.052
Create more awareness of the regulation		135	1		5	2.26	1.044
*c Note. 5 strongly agree (4.01–5.00), 4 agree (3.01–4.00), 3 neutral (2.01–3.00), 2 disagree (1.01–2.00), 1 strongly disagree (0.01–1.00)*							

Reliability Coefficient	Cronbach’s Alpha			N of Items			
0.909			5				

### 3.4 Trade barriers posed by the biosafety labelling regulations

As seen from the Likert scale mean, the response for all the questions (*Statements concerning trade barriers posed by the Biosafety labelling regulations*) falls in the range of (2.01–3.00), therefore it can be concluded based on the range used, the average response recorded for all those questions were neutral. Respondents were more leaning towards ‘different labelling regulations between trading partners’. This is a clear illustration of the respondents’ neutral position on the biggest threats to companies as a result of biosafety labelling regulations in Table 9.

**TABLE 9:** Trade barriers posed by the Biosafety labelling regulations.

Attributes		N	Minimum	Maximum		Mean	Std. Deviation
Different labelling regulations between trading partners		135	1	5		2.60	1.154
Lengthy and different timing approval processes between trading partners		135	1	5		2.45	1.124
Difficult in accessing food or feed risk assessment information from other countries		135	1	5		2.47	1.071
Unintentionally and unauthorized movement of unlabeled products		135	1	5		2.56	0.944
Inadequate segregation of unlabeled food and feed products (during production, storage, and transportation)		135	1	5		2.33	0.864
*c Note. 5 strongly agree (4.01–5.00), 4 agree (3.01–4.00), 3 neutral (2.01–3.00), 2 disagree (1.01–2.00), 1 strongly disagree* (*0.* ***01*** *–1.00*)							

Reliability Coefficient	Cronbach’s Alpha				N of Items		
0.733				5			

### 3.5 Eliminating trade barriers posed by the biosafety labelling regulations

Table 10 shows that with the reliability test of Cronbach’s Alpha coefficient (α = 0.847), means that the scale has good internal consistency. As seen from the Likert scale mean, the response for all the questions (Statements concerning trade barriers posed by the Biosafety labelling regulations) falls in the range of (2.01–3.00), therefore it can be concluded based on the range used, the average response recorded for all those questions were neutral. However, respondents have highlighted that integrating regional labeling regulations could eliminate trade barriers. The respondents have a neutral position on what needs to be done to eliminate trade barriers posed by the Biosafety labelling regulations.

**TABLE 10:** Eliminating trade barriers posed by the Biosafety labelling regulations.

Attributes	N	Minimum	Maximum	Mean	Std. Deviation
Integrate regional labeling regulations	135	1	5	2.74	1.178
Ban unlawful importing companies	135	1	5	2.12	1.127
Harmonize administration and enforcement of Namibian laws required for import	135	1	5	2.41	1.135
Improve public engagement for the newly introduced laws	135	1	5	2.24	1.272
*c Note. 5 strongly agree (4.01–5.00), 4 agree (3.01–4.00), 3 neutral (2.01–3.00), 2 disagree (1.01–2.00), 1 strongly disagree (0.01–1.00)*					

## 4 Discussions

### 4.1 Biosafety Act awareness

This result shows that individuals who are aware of the existence of the Biosafety Act, 2006 are less likely to agree with the labeling of Genetically Modified Food and Feed (processed) as required by the Biosafety labelling regulations as compared to individuals who are not aware of the existence of the Biosafety Act, 2006. The results might suggest that those that are aware of the existence of the Biosafety Act, 2006 do not agree with the labelling of GMO processed food and feed. Based on the findings of similar studies, a more plausible explanation is that there is an ongoing debate about whether genetically modified foods should be labelled, with some arguing that consumers should have the right to know everything about what’s in their food and others arguing that there is no evidence that such foods harm health and that labelling is not necessary (Yang and Chen, 2016). While past studies have concentrated on consumer protection with regard to GMOs (Monien and Cai, 2018), there have been less investigations on Food and Feed (processed) importers and exporters who are required to label Food and Feed (processed). According to studies conducted on importers and exporters, the economic implications of labelling GMOs are the stumbling block preventing them from those wishing to label them even when they are aware of the labelling legislation (Grebitus et al., 2018). As a result, the Food and Feed importers and exporters who participated in the study and are required to label the GMO Food and Feed are aware of the Biosafety Labelling regulation, and they are less likely to label GMOs as they might be concerned with the economic implications of labelling. These findings support Oh and Ezezika’s (2014) contention that such labelling may raise food costs, impeding attempts to meet food security requirements. Companies that develop GMO products are more likely to charge higher prices for their products such as GM seeds, with the intention of recouping their investments on research and development which can have a repel effect on the price of GMO product prices (Rutivi and Mugwagwa, 2009). The expenses of GMO review are determined not only by the criteria, but also by the length of time required to get a permit and the type of label on the products. These findings are supported by a study conducted in the United States as it indicated that when consumers were presented with conventional, organic, and non-GMO food options, the additional amount consumers were willing to pay for organic food was insignificant, indicating that producers would benefit economically by using a non-GMO label rather than organic certification (Grebitus et al., 2018). This suggests that being GMO-free may have a benefit, since it offers makers of GMO-free products with a significant motivation to market their products in this manner, putting less economic advantages on GMO-branded products. Due to the lack of data on the economic value of labeling in terms of cost and time of GMs due to the existence of the Biosafety Act, 2006 in Namibia, the results cannot confirm why those who are aware of the labelling regulations are less likely to agree with labelling of labeling of GMO Food and Feed because of economic value. Thus, the need for further studies in this regard.

The results shows that there is sufficient evidence to conclude that there is no significant difference in the agreeance of individuals who are aware of the labelling requirement under the Biosafety Act, 2006 with the labeling of Genetically Modified Food and Feed (processed) as required by the Biosafety labelling regulations. Twardowski and Małyska (2015) have indicated that stagnation in the implementation of biosafety frameworks is due to a lack of awareness.

### 4.2 The BSL adoption and application

Studies examining the companies applying to fulfil the requirements of the Biosafety Act are part of BSL adoption and application review. Many countries have several mechanism and requirements for BSL adoption and application, but few evaluate the companies applying to fulfil the requirements of the Biosafety Act with the purpose of improving the BSL adoption and application. This study assessed weather individuals whose companies applied to fulfil the requirements of the Biosafety Act, 2006 agreed with the statements regarding the labeling of genetically modified food and feed as required by the Biosafety labelling regulations in comparison to the companies who are unaware of the Biosafety labelling regulations. The Mann-Whitney test done indicates that individuals whose companies have applied to fulfil the requirements of the Biosafety Act, 2006 are more likely to agree with the statements regarding the labeling of genetically modified food and feed as required by the biosafety labelling regulations as compared to individuals whose companies have not applied to fulfill the requirements of the Biosafety Act, 2006.

The legal framework for labelling GMO products before placing the products on the market was necessary and thus the need for companies to apply to fulfil the requirements of regulations such as the Biosafety Act, 2006. The Mann-Whitney test agreeing that companies that have applied to fulfil the requirements of the Biosafety Act, 2006 are more likely to label GMO feed and food as required by the Biosafety labelling regulations is an indication of the agreement of BSL adoption and application by companies. The BSL adoption and application by companies is very important because the Biosafety Act was enacted so that all activities such as importation, production, release, and distribution are regulated to limit possible harmful consequences to the environment. According to the Government Gazette that deals with the Biosafety Act that regulates genetically altered products, non-compliance will lead to a fine not exceeding N$8,000 or imprisonment for a period not exceeding a 2 years or both.

The study found that individuals whose companies are experiencing problems in fulfilling requirements of the Biosafety Act, 2006 are less likely to agree with labeling of GM Food and Feed as required by the Biosafety labelling regulations in comparison to individuals whose companies are not experiencing problems in fulfilling requirements of the Biosafety Act, 2006. Existing regulations mandate labelling for both imported and domestically produced end goods (food and feed) to ensure that products are labelled in such a way that buyers are aware of the presence of GMOs in the products. But non-etheless, the Biosafety Act now requires permits to import, process, and transport such things, which were previously not necessarily due to the country’s long history of importing GMO food. Additionally, the law mandated that the permission application process be made public, and the public was allowed to express themselves whether such GMO permit should be given or denied. The Biosafety Council’s goal was to collect public inputs about GMO permits before determining whether to approve or reject such licenses. However, there is little to no research on the overall time it takes to fulfilling requirements of the Biosafety Act, 2006. However, the overall approval process for fulfilling requirements of the Biosafety Act, 2006 is longer than established 90 days to make a decision. The Cartagena protocol (article 11, 6b)) gives a maximum of 270 days) which is considering as the whole application to come to a decision.

### 4.3 Impact of labelling regulations

Results show that out of the ten statements concerning the biggest threats posed to companies regarding labelling regulations, the most significant threats posed by the Biosafety labelling regulations to companies are the increases in foreign competition and monopoly in the supply chain. A well-thought-out Biosafety labelling regulation should include many components that do not threaten companies, resulting in more good competition and less monopoly within the supply chain (Van der Walt, 2001). In the GMO labeling context, Kim et al. (2022) argue that a GMO label mandated by a regulatory body may send a negative signal that consumers should avoid a product with GM. Seeing that despite the presence of commercial farms in Namibia, the country has struggled to maintain the essence of food security thus striving through food imports, companies in Namibia would see the Biosafety labelling regulations as a threat towards foreign competition. A Biosafety labelling regulation that does not threaten companies in terms of ensuring that there are no impediments sch as foreign competition and monopoly in the supply chain towards companies can mitigate the impact of labelling regulations. However, Albert ed (2010) contends that, given the high level of competition in the global food market, consumer`s attitudes towards GM foods can heavily influence decisions made by farmers, commodity dealers, food manufacturers, and food retailers about whether to produce and market GM foods or use conventional varieties. Therefore, an understanding of the perceptions of, and likely reactions toward, genetically modified (GM) foods is crucial for decision making by both policymakers and biotechnology companies developers (Spence and Townsend, 2006). Institutions entrusted with enforcing the Biosafety labelling regulations should be strengthened to ensure that they understand the perceptions of, and likely reactions toward, genetically modified (GM) foods when making decisions to lessen the impacts of labelling regulations.

Results show that out of the six long-term effects for businesses caused by labelling regulations, the views on the threats posed by the Biosafety labelling regulations in terms of long-term consequences such as reduction in production, revenues, increase in production costs, loss of markets, retrenchment of employees and closing of the company are neutral. A neutral choice, according to DeMars and Erwin (2005), rewards responders who tilt slightly towards a favourable or unfavourable judgement. Thus, these findings can mean that the respondents were leaning slightly towards a favorable or unfavorable long-term effects for businesses because of labelling regulations stating that the labelling regulations are more neutral, but they are more geared towards leading to loss of markets with a mean of 2.53 and reduction in revenues with a mean of 2.59. In an ideal world, a well-thought-out mandatory labelling regulation would not jeopardize a company’s economic sustainability through market loss and revenue decrease (Van der Walt, 2001; Oh and Ezezika, 2014). Therefore, the long-term effects for business due to labelling regulations can have an impact on the Namibian economy.

In terms of revenue, food companies believe that the loss is due to the significantly high costs involved with mandatory labelling of GM products, as well as customer reaction towards GMOs. For example, research done in Kenya found that, while their food sector regarded it as vital to track GM products, many do not support labelling of GM products because of the additional costs and the likelihood of negative customer reactions (Bett et al., 2010). While studies (James, 2011; Oh and Ezezika, 2014; MacFarland and Yates, 2016; Strauss and Sax, 2016; Huffman and McCluskey, 2017) have been conducted, all former, demonstrating that mandatory labelling will incur additional costs that will eventually be passed on to the consumer, actual increases in food prices because of mandatory GM labelling have yet to be reported. Nevertheless, a 2001 research based on current EU legislation discovered that obligatory GM labelling adds an additional *per capita* yearly cost of around US$0.23 (Jones, 2001). A similar study done in Canada have shown that mandatory labelling in Canada would increase retail prices by 9%–10% (KPMG, 2000). Therefore, mandatory GM labelling law makes it important to fuel discussions about the costs of implementation in terms of the whole value chain as this have a significant impact on the companies dealing the GM products. According to Oh and Ezezika (2014), African governments including Namibia can benefit from reliable studies that allow them to examine the economic sustainability of regulating the mandate of GM labelling inside their individual nations before enacting a labelling law, as well as whose stakeholders may be impacted. In South Africa, Reddy (2017) showed that the direct cost increase of mandatory labelling to the consumer depends on many factors, but the average is calculated to be between 9% and 12%. This means that most of the market will suffer the expenses of GM labelling. More studies assessing the potential economic costs of mandatory labelling in Africa, however, are required, as most cost experiments pertaining to mandatory GM labelling are based on the experiences of countries other than Africa. Therefore, further case-by-case analyses of the economic consequences of mandatory GM labelling on Namibian food industry companies are required**.** Moreover, Namibia has developed and gazetted of genetically modified product list of GM transformed events that should first be approved for safe use even before consignments entering Namibian territory.

The policy recommendations towards the elimination of trade threats imposed by labelling regulations looked at wether the policy should reduce regulatory administrative burden, increase the competence of regulators, improve regulatory monitoring processes, improve dialogue between regulators and the industry or create more awareness of the regulation. The study findings were neutral on all five 5) statements even though the response was more geared towards the policy reducing regulatory administrative burdens and improving regulatory monitoring processes. This finding is consistent with studies that show the weaknesses within Namibia’s own Biosafety legal system (Geingos, 2018). Parker and Kirkpatrick (2012) highlighted that weakness within a legal system can be caused by regulatory administrative burdens and improving regulatory monitoring processes. However, administrative burdens in regulating GMO`s in Namibia are also rooted in the fact that even though Namibian regulatory framework made provision for a national GMO testing facility, this facility is still in the development phase and is not yet accredited. A GMO testing facility is essential for GM tracking, monitoring, and surveillance, as well as full compliance with national requirements (Kaiser et al., 2015). From the result, it can be inferred that limited regulatory monitoring processes such as that of the GMO testing laboratory require policy intervention in strengthening and reducing regulatory administrative burdens. However, Grechkina et al. (2019) concluded that the implementation of customs control over cross border movement of GMO foods improves efficiency and ensures the protection of the rights of citizens of the EAEU member States. Therefore, Namibia have developed and gazetted a genetically modified product list even though there is still a need to implement border control to sample GMO product consignments entering Namibia to ensure that permits granted are adhered to.

### 4.4 Trade barriers posed by the biosafety labelling regulations

The overall rating of the respondents shows that their response towards the trade barriers posed by the Biosafety labelling regulations is neutral. From the results, it can be inferred that the removal of trade barriers posed by the Biosafety labelling regulations in Namibia are more towards the different labelling regulations between trading partners and the unintentionally and unauthorised movement of unlabeled products. Trading of GMO products in Namibia as regulated by the Biosafety labelling regulations is affected by various contextual and administrative challenges such as poor regulatory frameworks. GMOs are widely available across the world, but they are also contentious and susceptible to regulatory scrutiny in terms of mandatory *versus* voluntary GMO labelling (Bullock and Desquilbet, 2002; Bhalerao and Kadam, 2010; Buah et al., 2021). Thus, different countries have developed and are implementing different GMO labeling policies to inform consumer choice if imported from countries with different percentage threshold levels of GMOs and labelling requirements. A good example is a difference in labelling requirements between the Biosafety Act, 2006 of Namibia and the South African Consumer Protection Act, 68 of 2008. When the GM content of a product exceeds 0.9%, it must be labelled as opposed to that which requires labelling when the GM content exceeds 5.0% (Charnovitz, 2000). Jacobs (2018) added that this GMO labelling regulations caused a severe negative impact on South Africans grain exporters to Namibia, it is expected to have very little impact on the South African grain industry as we export fairly small quantities to Namibia. This overlap between different country labelling may be an interesting area for future work as this may help to narrow the focus on finding a solution to the trade impacts of Biosafety labelling regulations. Hence the necessity to conduct further studies to critically review the way to ensure that labelling regulations between trading partners are married.

### 4.5 Eliminating trade barriers posed by the biosafety labelling regulations

The study looked at how trade barriers posed by the Biosafety labelling regulations can be eliminated based on six variables mainly in terms of integrating the regional labeling regulations, banning unlawful importing companies, harmonising administration and enforcement of Namibian laws required for import and improving public engagement for the newly introduced laws. The study responses were neutral but was more leaning towards favoring the integration of the regional labeling regulations. These findings are supported by Jacobs (2018) who found that South Africa’s labelling regulations is not in harmony with those of Namibia, with Namibia’s being more explicit than South Africa. However, the need to integrate labeling regulations has not only been a regional issue but it has been advocated at the international levels. There are also large differences in import-approval and marketing policies for GM food worldwide that can impact trade. Gruère (2006) noted that at the international level, harmonization efforts are led by the Codex Alimentarius Commission, the Cartagena Protocol on Biosafety (CPB), and the World Trade Organization (WTO). While internationally harmonized guidelines for safety approval have been finalized at the Codex Alimentarius, there is no clear consensus on labelling regulations for GM food, some of which could be found inconsistent with the WTO, and there is an increasing risk of conflicts between the CPB and the WTO. Even though there have been harmonization efforts at the international level, their consensus is more on safety approval, not on labeling. From a policy perspective, Gruère (2006) argues that there are three main spillover effects of national and international regulations on developing countries’ according to policymaking: 1) compliance with international agreements that do not necessarily correspond to domestic objectives, 2) the fear of export loss due to trade-related regulations implemented by the large importing countries, and 3) the trend toward harmonizing domestic regulations with those of the large importers. As a result, Namibia from a policy perspective must ensure that the Biosafety labelling requirements for GMOs are based on international standards while considering regional constraints and national preferences that give considerable productivity benefits to local businesses.

## 5 Recommendation

• Considering these conflicting viewpoints, the study suggests more research into how various GMO labelling legislation regimes affect the goods Namibian consumers choices. This will provide valuable input towards the Biosafety Act in terms of whether the sort of labelling being used affects customer choice.
• In practice, the research further suggests that the government raise public knowledge of GM foods through advertising, as well as boosting media coverage of GM safety and consumer choice through GM food labelling.
• To improve the adoption and application of the BSL, Namibia might learn from other countries e.g., South Africa and the EU that have already established processes.
• From a policy perspective, Namibia must ensure that the Biosafety labelling requirements for GMOs are based on best practices while taking into account regional constraints and national preferences that give considerable productivity benefits to local businesses
• Due to the lack of data on the economic value of labeling in terms of cost and time of GMOs due to the existence of the Biosafety Act, 2006 in Namibia, the results cannot confirm why those who are aware of the labelling regulations are less likely to agree with labelling of labeling of GMO products because of economic value. Thus, the need for further studies in this regard.
